# Hippocampal dysmetabolism contributes to cognitive loss in autoimmune encephalitis and focal temporal epilepsy

**DOI:** 10.3389/fneur.2025.1597928

**Published:** 2025-08-14

**Authors:** Olga Taraschenko, Lakshman Arcot Jayagopal, Audrina Mullane, Kyle Greenman, Matthew White, Hesham Ghonim, Shelley Lee, Rana Khalil Zabad, Tracy Jasinski, Mariano Uberti

**Affiliations:** ^1^Department of Neurological Sciences, Division of Epilepsy, University of Nebraska Medical Center, Omaha, NE, United States; ^2^Department of Neurological Sciences, Division of Neuropsychology, University of Nebraska Medical Center, Omaha, NE, United States; ^3^Department of Radiology, University of Nebraska Medical Center, Omaha, NE, United States; ^4^Department of Neurological Sciences, Division of Neuroimmunology, University of Nebraska Medical Center, Omaha, NE, United States

**Keywords:** autoimmune encephalitis, temporal lobe epilepsy, cognitive loss, memory deficits, cerebral metabolism, myo-inositol, magnetic resonance spectroscopy

## Abstract

**Introduction:**

Autoimmune encephalitis (AE) is associated with severe cognitive disability. Brain metabolic dysfunction has been linked to encephalopathy in neurodegenerative disorders; however, its role in the development of cognitive loss in AE has not been studied. We hypothesized that cognitively impaired patients with AE will demonstrate altered brain metabolism and immune activation, and these measures will correlate with cognitive scores.

**Methods:**

The hippocampal and cortical metabolites related to neuronal integrity, oxidative metabolism, and glial activation were assessed using single-voxel proton magnetic resonance spectroscopy (1H-MRS) in patients with AE, non-lesional temporal lobe epilepsy (TLE) and control subjects. Metabolite levels were correlated with neuropsychological test scores.

**Results:**

We recruited patients with post-acute AE (*n* = 12), non-lesional TLE (*n* = 12), and control subjects (*n* = 11). Subjective cognitive complaints were reported by 83.3% of AE and all TLE patients. AE patients had fewer seizures and used fewer anti-seizure medications than TLE patients (*p* = 0.04, *t*-test and *p* = 0.03, *post-hoc* test). On neuropsychological testing, moderate and severe cognitive impairment was revealed in 58.3% of patients with AE and 41.6% of patients with TLE. Hippocampal myo-inositol (M-Ins) concentrations were higher in patients compared to control subjects, with a trend toward increase in AE and TLE relative to control (*p* = 0.046, ANOVA; *p* = 0.09 and *p* = 0.07 for AE and TLE vs. control, respectively; *post-hoc* tests). The concentration of creatine (tCr) and total choline (tCho) were significantly higher in patients with TLE compared to the controls (tCr: *p* = 0.007; tCh: *p* = 0.04; *post-hoc* tests). Elevated M-Ins in AE was associated with better attention but worse memory recognition scores (*R*^2^ = 0.38, *p* = 0.04 and *R*^2^ = 0.50, *p* = 0.02, respectively); higher tCr levels correlated with faster processing speed (*R*^2^ = 0.38; *p* = 0.04). The higher concentrations of tCr, tCho, and M-Ins in TLE have selectively correlated with worse measures of attention, processing speed, language, and memory.

**Conclusions:**

Although AE and TLE patients report similar cognitive issues, their hippocampal metabolic signatures differ. The disease-specific changes in the measures of hippocampal inflammation and neuronal integrity can inform trajectories for cognitive recovery and be targeted therapeutically.

## 1 Introduction

Autoimmune encephalitis (AE) is an acute severe non-infectious brain inflammation associated with autoantibodies against cell surface proteins and intracellular targets (1). The AE syndromes share common manifestations of precipitous cognitive decline, psychosis, abnormal movements, and new-onset seizures (2). Once the acute life-threatening symptoms are controlled, patients face persistent cognitive loss which can continue for years and can be life-long (3, 4). Cognitive deterioration, particularly memory and language dysfunction results in loss of productivity and greatly impacts patients' quality of life. Considering that the incidence of AE has been consistently on the rise (5) and the success of existing therapies is modest, new therapeutic targets to preserve cognition are urgently needed.

The most common AE syndrome, anti-NMDA receptor (NMDAR) encephalitis, targets the hippocampus and other cortical areas. It leads to executive dysfunction and memory difficulties that persist for more than two years after the resolution of the acute phase (6, 7). The incomplete recovery of cognitive function is particularly detrimental to young patients who have a very high incidence of anti-NMDAR encephalitis as it limits their employment and other social opportunities. The anti-LGI1-antibody-associated encephalitis is another prevalent AE syndrome that affects limbic temporal lobe areas leading to severe permanent deficits in anterograde and episodic memory functions in up to one-third of patients (8, 9). Lastly, the anti-glutamic acid decarboxylase (GAD65)- associated AE which also has a predilection to the limbic areas, results in long-lasting executive dysfunction and prominent amnesia in recovering patients (10, 11). Chronic memory impairment is also present in AE syndromes for which the antibody targets have not been yet identified (i.e., antibody-negative AE) (12) suggesting that the pathophysiology of chronic cognitive loss could be shared among encephalitis syndromes.

The distinct pathophysiological mechanisms mediate the acute effects of autoantibodies supporting the unique clinical presentations of these syndromes. However, the chronic sequala of AE which persists upon removal of antibodies, could share the same pathophysiology. One potential contributor to the cognitive loss in AE could be chronic immune activation which may persist despite the immunotherapies. Neuroinflammation and brain metabolic dysfunction have been recently looked at in the context of cognitive dysfunction in patients with mild cognitive impairment (MCI) and Alzheimer's Disease (AD) (13, 14). However, it is not clear whether it contributes to cognitive loss in AE. In the present study, we used single-voxel proton magnetic resonance spectroscopy (^1^H-MRS) semi-Localized by Adiabatic Selective Refocusing (semiLASER) sequence (15) to assess relevant metabolite measures in AE and correlate them with patients' cognitive scores obtained during targeted neuropsychological evaluation. Compared to conventional sequences, semi-LASER provides improved accuracy and sensitivity in measuring metabolite concentrations, especially at higher field strengths and in brain structures with less uniform magnetic fields, such as the hippocampus (16). To determine if changes in metabolites could be influenced by the effects of anti-seizure medications (ASMs) and to establish the role of seizures in these changes, we compared the metabolic profile of patients with AE to that of patients with non-lesional temporal lobe epilepsy (TLE) and control subjects. Our focus was on the hippocampal-based cognitive measures and regional ^1^H-MRS markers of neuronal integrity, oxidative stress, and immune activation (17). We hypothesized that cognitively impaired patients with AE will demonstrate altered metabolism and enhanced immune activation in the hippocampus and these measures will correlate with their performance on cognitive tests.

## 2 Materials and methods

### 2.1 Participants

The study was approved by the Institutional Review Board (IRB) at the University of Nebraska Medical Center (UNMC); all the participants signed the informed consent. This prospective non-interventional cohort study followed the guidelines outlined in the Strengthening the Reporting of Observational Studies in Epidemiology (STROBE) document (Supplementary Table 1). Male and female patients with a history of definite antibody-positive AE and probable antibody-negative AE established based on the accepted diagnostic criteria (2) were recruited from the Autoimmune Seizure Clinic and Neuroimmunology Clinic at UNMC between April 1, 2023, and June 30, 2024. The inclusion criteria were clinical recovery from acute encephalitis and eligibility for magnetic resonance imaging (MRI). Patients of both sexes with TLE without structural lesions on clinical MRI and subjective complaints of cognitive impairment were recruited from the epilepsy clinic at UNMC, the level 4 epilepsy center. The diagnosis of focal epilepsy was confirmed with ictal electroencephalography (EEG) recordings. The control subjects without a history of neurological diseases matched to the patients with respect to age, sex, and educational level were recruited from the Brain and Mind Health Registry at UNMC. The group size was determined based on the findings from the pilot study.

### 2.2 Clinical assessments

The demographic and clinical data of patients were extracted from the charts. The diagnosis of AE was confirmed by two neurologists experienced in autoimmune seizure disorders (O.T. and L. A.) (2); the disease duration was defined as the interval between the onset of symptoms and the time of the research imaging and cognitive tests. The patients' definite encephalitis type was supported by the antibody status of their cerebrospinal fluid (CSF) or serum (2). The clinical symptoms, seizure status, EEG and imaging findings at onset as well as utilization of ASM and immunotherapy agents were recorded at the last follow-up visit (Supplementary Tables 3, 4).

### 2.3 Neuropsychological evaluation

The targeted neuropsychological evaluation was conducted by a clinical neuropsychologist (A.M.) or neuropsychology postdoctoral fellow (K. G.) and included the assessment of attention, processing speed, executive functioning, language, visuoperception, memory, and recognition functions (Supplementary Table 2). The examiners were blinded to the disease status of the participants. Participant responses were scored using normative data to account for age, sex, and educational variability; the normative scores were standardized into z-scores for analysis. Additionally, each test and selected task components were separated into their respective domains and the mean z-score was calculated for each domain generating an estimate of domain functioning. Abnormal performance was defined as one or more scores falling at least 1.5 standard deviations (SD) below the mean (*z* score = ≤ -1.5) (6). Further, a cognitive impairment score, or a composite score, was assigned based on the overall number of affected domains, which was defined as mild (1 domain affected), moderate (2–3 domains affected), or severe (≥4 domains) (6). All participants were screened for depression, anxiety, and insomnia using the self-report questionnaires (Supplementary Table 2).

### 2.4 Imaging studies

#### 2.4.1 MRI and single-voxel 1H-MRS acquisition

**Figure 1:**
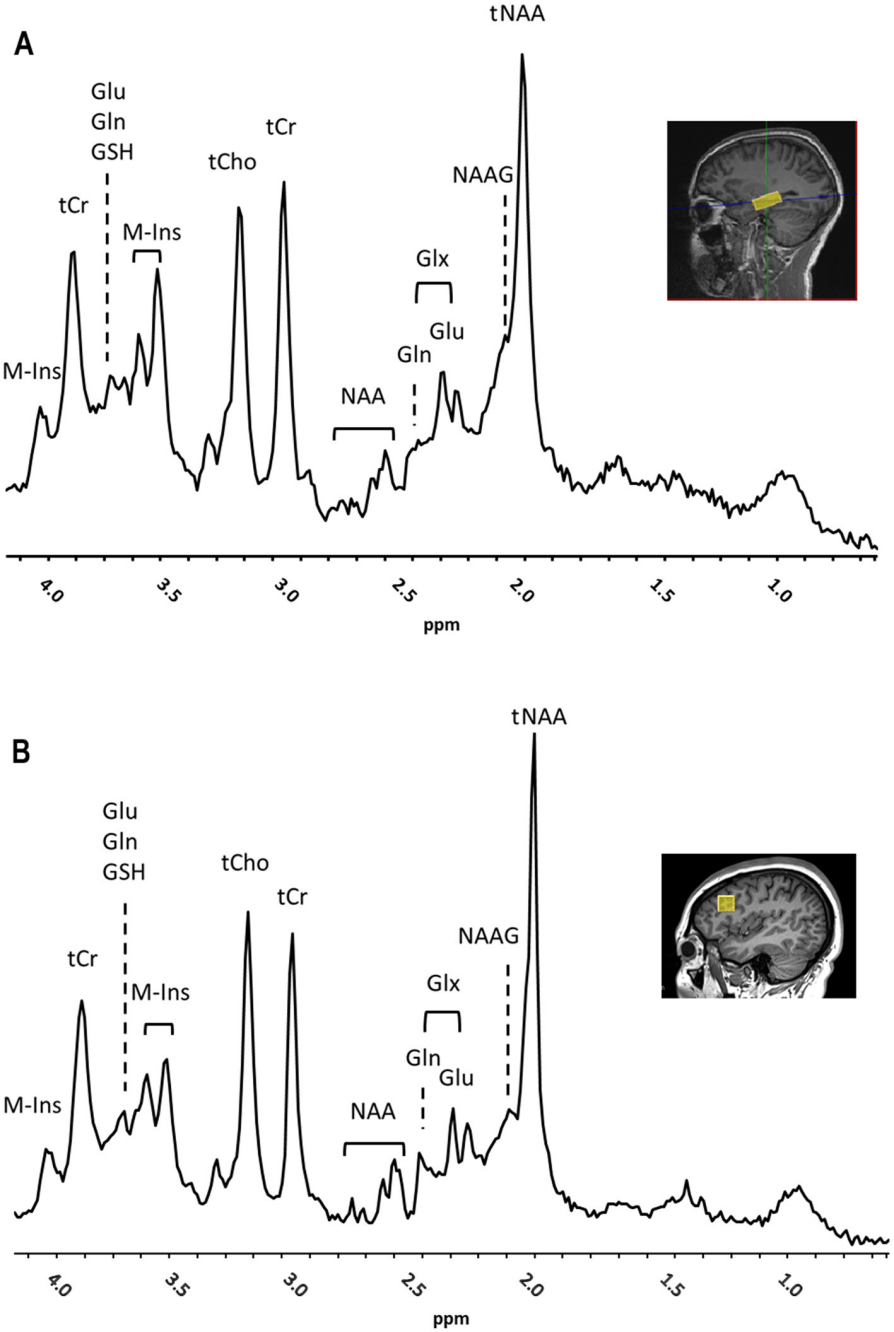
Representative ^1^H-MRS spectra and placement of volume of interest (VOI) (yellow box) for right hippocampus **(A)** and left prefrontal cortex **(B)** showed on a sagittal MRI (**insets**) from a subject with autoimmune encephalitis (AE). Main peaks for total N-acetyl aspartate and N-acetyl–aspartyl–glutamate (tNAA), total choline (tCho), total creatine (tCr), myo-Inositol (M-Ins), glutamate (Glu), glutamine (Gln), Glx, Glu and Gln, and glutathione (GSH), are labeled. Chemical Shift was expressed in parts per million (ppm).

All data were obtained on a Siemens Prisma VE11c 3.0 T scanner (Siemens Healthcare, Erlangen, Germany) equipped with 80 mT/m at 200 T/m/s high-performance gradients with high-order shim and highest density 32-channel head coil during one or two sessions within 1–2 weeks from the cognitive testing. Total scanning time was approximately 90 min. The 3D T1-weighted magnetization-prepared rapid gradient-echo sequence (MPRAGE, TR/TE = 2300/2.29 ms, FOV = 240 mm, 192 slices, voxel size = 0.9 × 0.9 × 1.0 mm) was used to acquire anatomical reference images used for volume of interest (VOI) placement. Single-voxel ^1^H-MRS data was acquired on the left and right hippocampi (VOI: 30 × 12 × 12 mm^3^; ≈4.3 ml) and left prefrontal cortex (VOI:16 × 16 × 16 mm^3^; ≈4.1 ml) using a short TE semiLASER sequence (TR/TE = 5000/30 ms, spectral points = 2048, spectral width = 6002.4 Hz, averages = 128) with 2 ms excitation and 4 ms Frequency Offset Corrected Inversion (FOCI) refocusing pulses (18–20) (Figure 1). Water suppression was achieved using Variable Power and Optimized Relaxation (VAPOR) delays (21). B_0_ shimming was performed with Fast, Automatic Shimming Technique by Mapping Along Projections (FASTMAP) (22). B_1_ pulses were manually calibrated for each VOI. Outer volume suppression (OVS) was used to suppress contamination from signals originating from outside the VOI. In addition to metabolite spectra (*n* = 128), water reference scans (*n* = 4) were acquired. Center frequency was 2.67 ppm for all metabolites and 4.67 ppm for water scans. Spectra were saved both as the sum and as single transients. The image analysis was performed by a neuroradiologist (M.W.) and a computational scientist with expertise in MRS (M.U.); all investigators were blinded to the participant's clinical status.

#### 2.4.2 ^1^H-MRS analysis

The metabolites that reflected the neuronal health and integrity (23), brain oxidative metabolism (24), and immune activation (25) were measured in the left and right hippocampus and left prefrontal cortex (Figures 1A, B). The total mean concentration of total N-acetyl-aspartate [tNAA: NAA and N-acetyl–aspartyl–glutamate (NAAG)], choline-containing compounds (tCho), creatine and phosphocreatine (tCr), glutamate (Glu), glutamine (Gln), *myo*-inositol (M-Ins) and glutathione (GSH) were calculated. The MRS data were preprocessed using MRSpa software in Matlab (26). For each VOI, the resulting 128 transients of the suppressed free induction decay (FID) signal were processed as follows: (1) Eddy current correction was applied using the unsuppressed “water” signal, (2) motion-corrupted transients were removed to ensure data quality, (3) frequency shift and phase variations in each transient were corrected, and (4) all corrected transients were added to generate a final FID. Average brain tissue water content was determined, assuming 81% of water in gray matter (GM), 71% in white matter (WM) and 100% in CSF using calculated within-VOI fractions of GM, WM, and CSF obtained from tissue segmentation (27, 28). The within-VOI average brain tissue water (BWC) and the mean CSF fraction were used to correct metabolite concentrations (29, 30). Fitting was carried out with a linear combination modeling in the LCModel software using a simulated basis set composed by 20 metabolite and 1 macromolecule baseline signals as pre-knowledge, and accounting for tissue type composition in the VOI (31, 32). Spectral analysis was conducted over the 0.5–4.2 ppm range, with zero- and first-order phases set to zero. Concentrations of metabolites were calculated relative to the concentration of water in the VOI in absolute units (in μmol/g). For each VOI, the signal-to-noise ratio (SNR) was measured as a ratio of the height of NAA peak at 2.02 ppm and root mean square of the noise (at ≈ [−2 0] ppm) in the metabolite spectra. In addition, the linewidth was calculated as full width at half maximum (FWHM) in the water spectra. SNR and FWHM were used as indicators of spectral quality; the data points were included if SNR ≥ 12 and FWHM < 0.1 ppm. Moreover, for each of the metabolite concentration measurements, the goodness of fit threshold for inclusion in the analysis (quantification reliability) was determined by a mean Cramer-Rao Lower Bounds (CRLB) ≤ 30% SD. One patient data set was excluded from further analysis due to poor spectra quality caused by movement. All MRS data were confirmed to meet the quality criteria based on the experts' consensus recommendations (16).

#### 2.4.3 Segmentation

Tissue segmentation of within-VOI brain volume was performed using SPM12 software (33, 34) and a custom MATLAB script in order to determine and compare VOI tissue composition of GM, WM, and CSF between groups, as described in previous studies (28). The calculated within-VOI BWC and fraction of CSF were used to correct metabolite concentration values for each group.

### 2.5 Statistical analysis

The quantitative variables and categorical variables were compared using ANOVA and Fisher exact tests, respectively. The *post hoc* Tukey tests adjusted for pairwise comparisons were used when appropriate (GraphPad Prism 10.2.3, Boston, MA). The proportions of patients were compared using Fisher exact tests. The relationships between cognitive measures and brain metabolites were studied using linear regression analysis; Pearson correlation coefficients were computed. The fractions of WM, GM and CSF in the MRS VOI were compared between the control, AE and TLE groups using multivariate ANOVA. The SNR and FWHM values were compared between the groups using ANOVA.

## 3 Results

### 3.1 Patient characteristics

Two and one control subjects were excluded because of the abnormal cognitive scores and unacceptable ^1^H-MRS data quality, respectively yielding 11 subjects. Additional patients with cognitive complaints and AE or TLE were recruited (*n* = 12 per group). There were no significant differences concerning age, sex, handedness, and years of education between the three groups (Table 1). The durations of AE and TLE were comparable although it tended to be shorter in AE (*p* = 0.06; *t*-test; Table 1). The median seizure counts and ASM usage were significantly lower in AE compared to the TLE group (*p* = 0.04, *t*-test and *p* = 0.03, *post-hoc* test). The non-ASM usage was higher in the TLE group compared to the control (*p* = 0.02, *post-hoc* test; Table 1).

**Table 1:** Demographic and clinical characteristics of patients with autoimmune encephalitis (AE), temporal lobe epilepsy (TLE), and control subjects.

**Patient characteristics**	**Control**	**AE**	**TLE**	***p*-value**
Age, years (SD)	33.9 (13.9)	38.7 (16)	45 (16.3)	0.24
Sex (percent female)	54.5	66.7	66.7	0.82
Handedness (percent right)	81.8	88.3	83.3	0.99
Education, years (SD)	15.6 (2.9)	14.2 (1.5)	14.7(1.9)	0.27
Disease duration, months (SD)	n/a	67 (59)	119.3 (105.8)	0.16
Seizure counts/year (CI)	n/a	0 (0-2)	12 (2–48)	**0.04**
ASM usage (CI)	n/a	1 (0-3)	3 (2–4)	**0.03**
Immunotherapy usage (CI)	n/a	1 (1,2)	n/a	
Other medication usage (CI)	1 (0-3)	1.5 (0-5)	6 (4–8)	0.01^*^

The data are the group means (age, years of education, disease duration) or medians (seizure counts and medication usage). SD, standard deviation; CI, 25–75% confidence interval; ASM, anti-seizure medications.

* *p* < 0.05, control vs. TLE, *post-hoc* tests. Values in bold denote significant differences, AE vs. TLE.

The mean age (SD) of patients with AE was 38.7 (16) years. The anti-NMDAR encephalitis was diagnosed in 5 patients while anti-LGI-1 encephalitis and GAD-65 encephalitis were present in 1 and 2 patients, respectively (Supplementary Table 3). Four patients had probable AE with no detectable antibodies (2). Among those, 3 patients have recovered from the new onset refractory status epilepticus (NORSE). The paraneoplastic etiology was ruled out in all patients with appropriate diagnostic tests. The mean disease duration from the onset of first symptoms to the time of study in patients with AE was 67 (59) months. The temporal lobe appearance in MRI was unremarkable in 10 patients but showed decreased size and increased signal intensity in bilateral hippocampi in one patient and decreased volume of the left amygdala in another patient (Supplementary Table 3). The annual seizure frequency (median; confidence interval, CI) was 0 (0–2) and the ASM usage was 1 (0–3). Patients were receiving immunotherapy with 1 (1,2) agents, including rituximab, intravenous immunoglobulin, and anakinra (Supplementary Table 3). All patients were living independently while 6 were employed and 1 was studying in college.

Patients with TLE had a mean age of 45 (16.3) years and suffered from epilepsy on average for 119.3 (105.8) months (Table 1). The disease was deemed to be medically refractory in 9 out of 12 (75%) patients (Supplementary Table 4). The MRI did not reveal structural abnormalities in the temporal lobes of patients with TLE except for a suspected left hippocampal cyst in one patient and bilateral temporal meningoceles in another patient (Supplementary Table 4). Patients reported a median annual count of 12 (2–48) seizures with various semiology and were on 3 (2–4) ASMs (Table 1).

### 3.2 Cognitive deficits, mood disturbances, and sleep disorders

**Figure 2:**
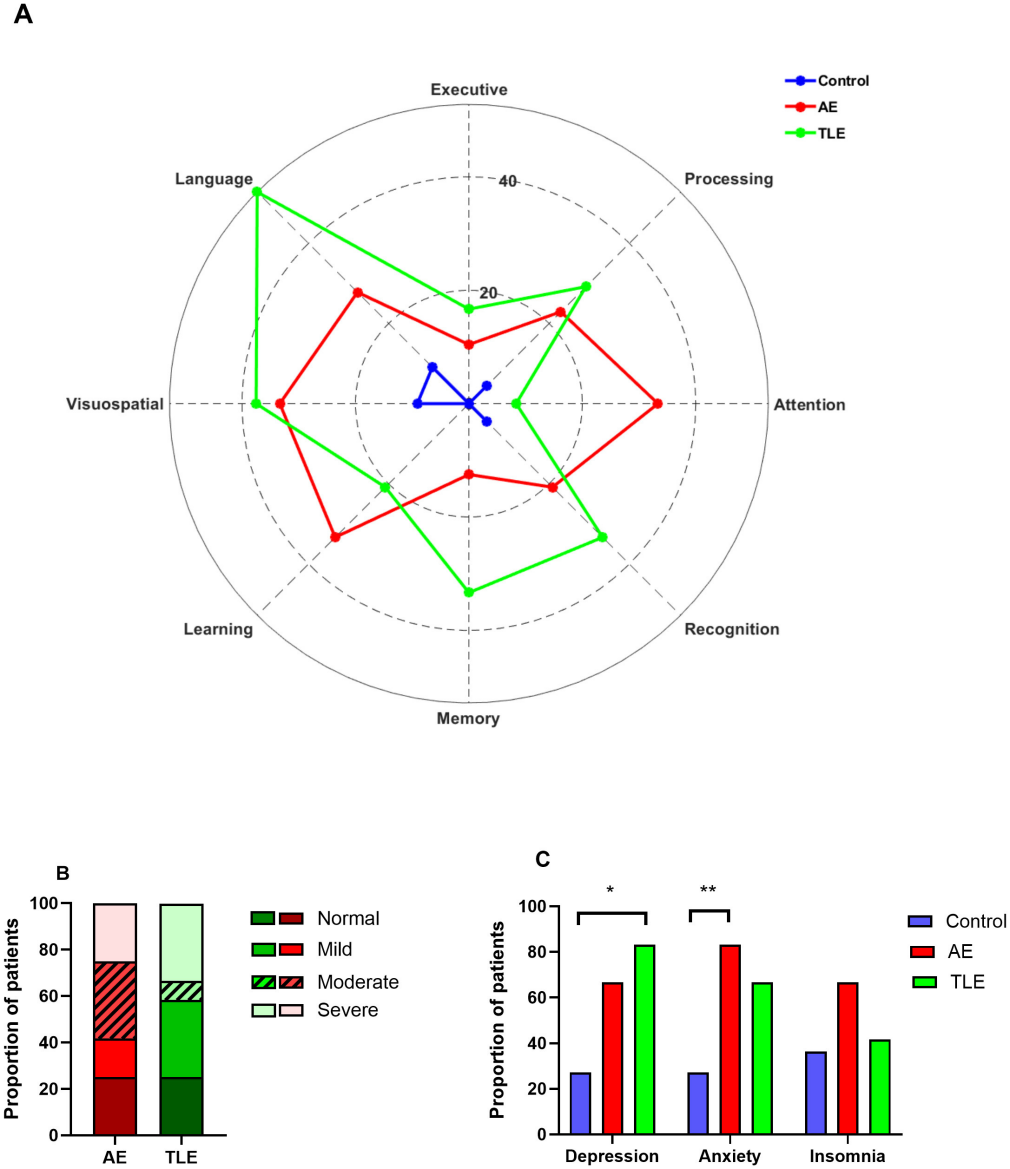
Cognitive, psychiatric and sleep disturbances in patients with autoimmune encephalitis (AE), temporal lobe epilepsy (TLE). **(A)** Distribution of patients with abnormal cognitive performance among AE, TLE and normal subjects. The data are mean proportions of patients with abnormal scores in each cognitive domain. The abnormal scores were defined as *z* ≤ −1.5. **(B)** The spectrum of the cognitive impairment severity in patients with AE and TLE. The data are the proportions of patients with different severity categories in two groups. **(C)** Psychiatric co-morbidities and insomnia profiles of patients with AE, TLE, and control subjects. The data are the proportions of patients. *, *p* < 0.05; **, *p* < 0.01; Fisher exact tests.

Ten patients with AE (83.3%) and all patients with TLE reported subjective complaints of cognitive impairments. Upon formal testing, patients with AE and TLE showed deficits in multiple tests designed to evaluate attention, processing speed, executive function, language, learning, memory, and recognition (Figure 2A). Abnormal performance in patients with AE was most apparent on the tasks of attention, processing speed, language, visuospatial skills, learning, and recognition. The deficits in the TLE group were most prominent in the processing speed, language, visuospatial skills, memory, and recognition (Figure 2A). To determine the distribution of the overall cognitive performance in both groups, a composite qualitative severity score was assigned based on the number of domains that were deficient. The proportions of patients with normal performance and mild, moderate, and severe impairment were 25, 16.7, 33.3, and 25% in AE and 25, 33.3, 8.3, and 33.3% in the TLE group, respectively (Figure 2B) There were no significant differences between the distributions of these patients (*p* = 0.3; Fisher exact test).

Given that psychiatric co-morbidities and insomnia are exceedingly prevalent in AE (9), we determined how these disorders have affected our populations. Overall, the proportions of patients with depression and anxiety differed significantly among the three groups (*p* = 0.03 and *p* = 0.03, respectively; Fisher exact tests). However, the proportions of patients with insomnia were comparable in three groups (*p* = 0.34, Fisher exact test). Specifically, patients with AE reported anxiety more often than control subjects but experienced depression and insomnia as frequently as control subjects (*p* = 0.01; *p* = 0.1; *p* = 0.22, respectively; Fisher exact tests; Figure 2C). On the other hand, patients with TLE had higher rates of depression compared to the control subjects but had similar rates of anxiety and insomnia (*p* = 0.01; *p* = 0.1 and *p* = 0.99, respectively; Figure 2C). The rates of depression, anxiety and insomnia were comparable in patients with AE and TLE (*p* = 0.64, *p* = 0.64; *p* = 0.41, respectively).

### 3.3 Brain metabolite measures

**Figure 3:**
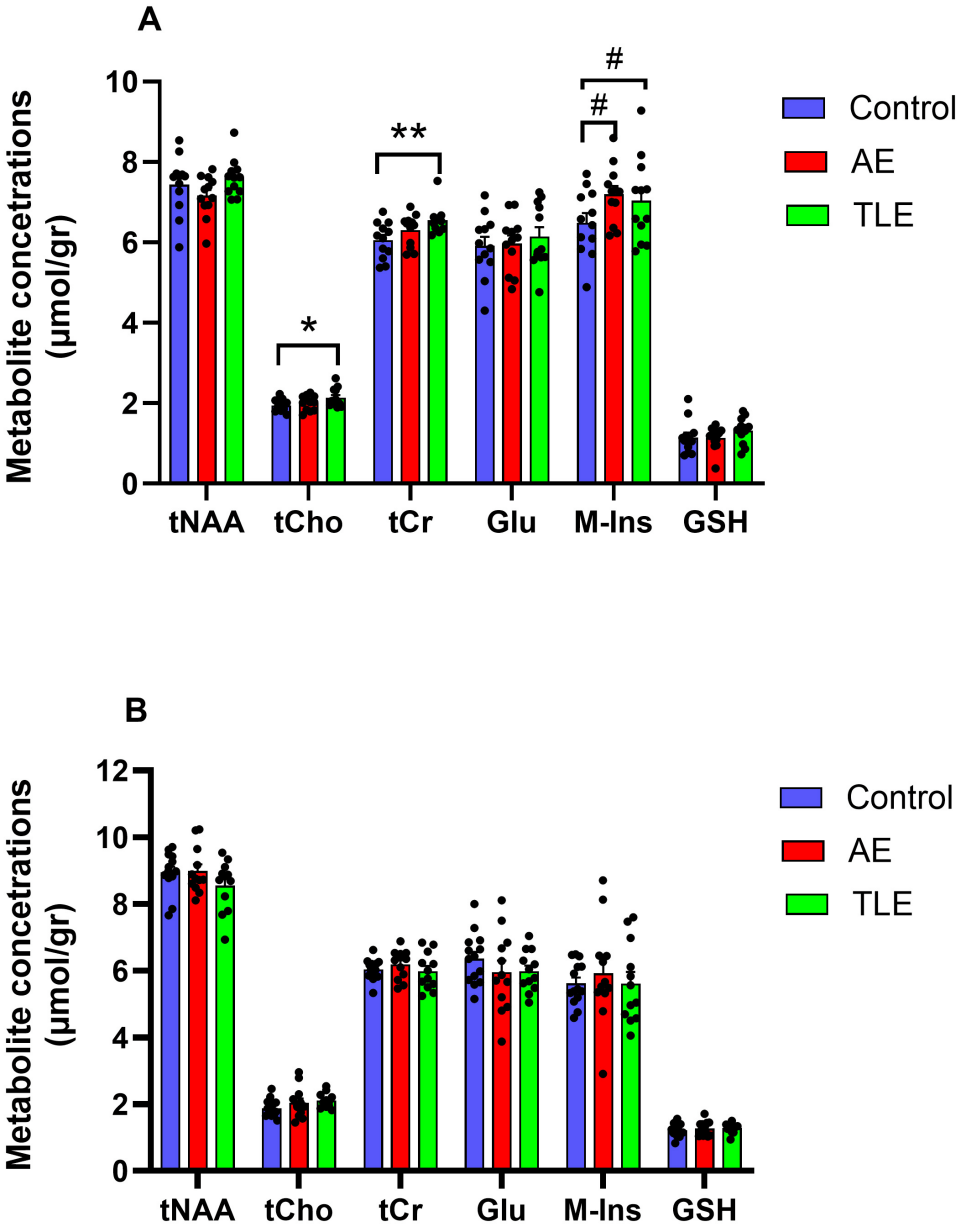
Metabolite profiles of patients with autoimmune encephalitis (AE), temporal lobe epilepsy (TLE), and control subjects. **(A)** The concentrations of *myo*-inositol (M-Ins) in the hippocampus were significantly higher in patients with AE compared to the control subjects while concentrations of total choline (tCh), total creatine (tCr), and *myo*-inositol (M-Ins) were significantly higher in patients with TLE compared to the control subjects. tNAA, total N-acetyl aspartate, and N-acetyl–aspartyl–glutamate; Glu, glutamate; GSH, glutathione. **(B)** The concentrations of metabolites in the cortex were comparable in all three groups. **, *p* < 0.01; *, *p* < 0.05; #, 0.5 ≤ *p* ≤ 0.1; *post hoc* tests. The data are mean concentrations (μmol/g) ± standard errors of the mean (SEM).

All metabolites of interest were reliably quantified, except for Gln, which had a mean CRLB > 30% and was excluded from the analysis (Supplementary Figures 2A, B). The concentrations of metabolites (in μmol/g) in the left and right hippocampus were comparable (tNAA: *p* = 0.42, tCho: *p* = 0.92, tCr: *p* = 0.30, Glu: *p* = 0.32, M-Ins: *p* = 0.36, GSH: *p* = 0.94; *t*-tests). Therefore, the mean values were used. The SNR and linewidth were of high quality and consistent across groups. The spectral SNRs (mean and SD) were 73.77 ± 11.70 and 74.27 ± 13.89 for the right and left hippocampus VOI, respectively. The spectral SNR for the cortical VOI was 165.13 ± 31.41. The FWHM values were 10.66 ± 1.44, 10.68 ± 1.60 and 7.33 ± 1.52 Hz for the right hippocampus, left hippocampus and cortical VOI, respectively. There were no differences between the SNR and FWHM values in the hippocampus of the control, AE, and TLE groups (*p* = 0.26 and *p* = 0.12, respectively; ANOVA). Furthermore, there were no differences in these values in the cortex of the control, AE, and TLE groups (*p* = 0.68 and *p* = 0.61, respectively; ANOVA). The average within-VOI tissue composition – fractions of GM, WM, and CSF- were similar among groups in both hippocampus (Control vs. AE: *p* = 0.50, Control vs. TLE: *p* = 0.57, AE vs. TLE: *p* = 0.43; multivariate ANOVA Wilk's Lambda test) and cortex (Control vs. AE: *p* = 0.54, Control vs. TLE: *p* = 0.40, AE vs. TLE: *p* = 0.94; multivariate ANOVA Wilk's Lambda test). Measured SNR, FWHM and within-VOI tissue composition values are shown in Table 2. The tCho tended to differ between the three comparison groups and were significantly higher in the TLE group compared to the control (*p* = 0.05, ANOVA; *p* = 0.04; *post-hoc* tests; Figure 3A; Supplementary Table 5). The concentrations of tCr in the hippocampus were significantly different in patients with AE, TLE, and normal subjects and were higher in the TLE groups compared to the control group (*p* = 0.01, ANOVA; *p* = 0.007; *post-hoc*; Figure 3A; Supplementary Table 5). The concentrations of M-Ins were significantly higher in both patient groups compared to the control group (*p* = 0.048 and *p* = 0.046, respectively; ANOVA). However, *post-hoc* analysis showed only a trend toward higher levels in AE and TLE compared to controls (*p* = 0.09 and *p* = 0.07; *post hoc* tests, respectively). On the other hand, the concentrations of tNAA, Glu, and GSH in the hippocampus were similar all comparison groups (*p* = 0.19; *p* = 0.82; *p* = 0.36; Figure 3A). The concentrations of tNAA, tCho, tCr, Glu, M-Ins, and GSH (in μmol/g) in the cortex were similar in all three groups (*p* = 0.25; *p* = 0.07; *p* = 0.44; *p* = 0.43, *p* = 0.71, and *p* = 0.77, respectively; Figure 3B; Supplementary Table 5).

**Table 2:** Measures of spectral quality and MRS-voxel tissue composition (mean ± SD).

**Group**	**SNR**	**FWHM (Hz)**	**GM (%)**	**WM (%)**	**CSF (%)**	**BWC (%)**
**Hippocampus**						
Control	72.4 ± 12.0	11.0 ± 1.4	68.3 ± 7.6	28.5 ± 8.0	3.0 ±2.0	78.6
AE	75.0 ± 12.5	10.6 ± 1.5	64.5 ± 19.9	32.4 ± 11.1	3.0 ± 1.4	78.3
TLE	75.1 ± 14.1	10.3 ± 1.6	69.2 ± 18.7	27.6 ± 8.9	3.2 ± 1.7	78.9
**Cortex**						
Control	176.4 ± 25.4	7.4 ± 0.6	12.6 ± 7.3	87.0 ± 7.6	0.4 ± 0.5	72.4
AE	168.0 ± 35.6	7.9 ± 1.0	13.7 ± 11.6	85.2 ± 12.1	1.0 ± 1.3	72.6
TLE	168.2 ± 30.2	7.3 ± 0.76	11.4 ± 4.7	87.7 ± 4.5	1.0 ± 0.7	72.5

SNR, signal-to-noise ratio; FWHM, full width half maximum; GM, gray matter; WM, white matter; CSF, cerebrospinal fluid; BWC, average brain tissue water content; AE, autoimmune encephalitis; TLE, temporal lobe epilepsy.

### 3.4 Correlations of brain metabolite concentrations with cognitive performance

**Figure 4:**
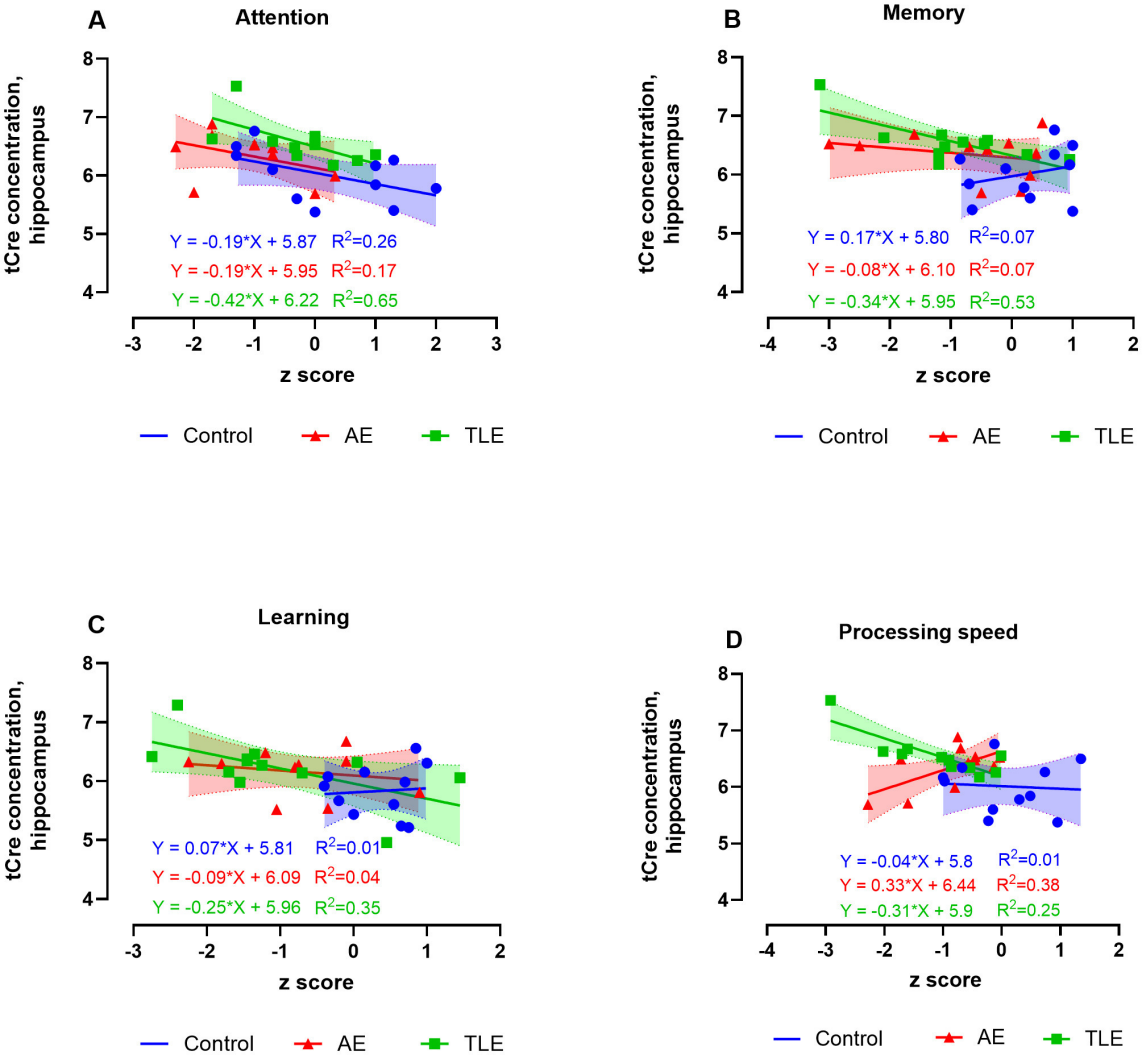
Relationship between the ^1^H-MRS measures of hippocampal total creatine (tCr) and cognitive performance in patients with autoimmune encephalitis (AE), temporal lobe epilepsy (TLE), and control subjects. The levels of tCr negatively correlated with scores attention, memory, and learning scores of attention in TLE **(A, C, D)**, and positively correlated with the scores of processing speed in AE **(B)**. The data are concentrations of metabolite (μmol/g) and mean standardized z scores for each cognitive domain.

One patient with AE was suffering from color vision deficiency and was an outlier in the measures involving visual function; therefore, he was removed from correlation analyses. Since we found the overall differences in the levels of tCho in patients with AE, TLE, and control subjects and based on the previous findings linking choline, creatine, and *myo*-inositol to cognitive function in neurodegenerative disorders (25), we examined the correlation of signals from these metabolites with cognitive measures in all three groups. There were no correlations between the hippocampal tCho levels and cognitive scores in AE or control subjects. However, in TLE, tCho negatively correlated with the measures of attention (*R*^2^ = 0.64, *p* = 0.002), processing speed (*R*^2^ = 0.56, *p* = 0.005), and language (*R*^2^ = 0.44, *p* = 0.02) such that higher metabolite levels were associated with worse cognitive performance (Supplementary Figures 1A–C; Supplementary Table 6). The hippocampal tCr levels negatively correlated with scores of attention, memory, and learning in TLE (*R*^2^ = 0.65, *p* = 0.002; *R*^2^ = 0.53, *p* = 0.007; *R*^2^ = 0.35, *p* = 0.04, respectively, Figures 4A–C; Supplementary Table 6). However, the tCr levels have positively correlated with measures of processing speed in AE (*R*^2^ = 0.38; *p* = 0.04; Figure 4D).

**Figure 5:**
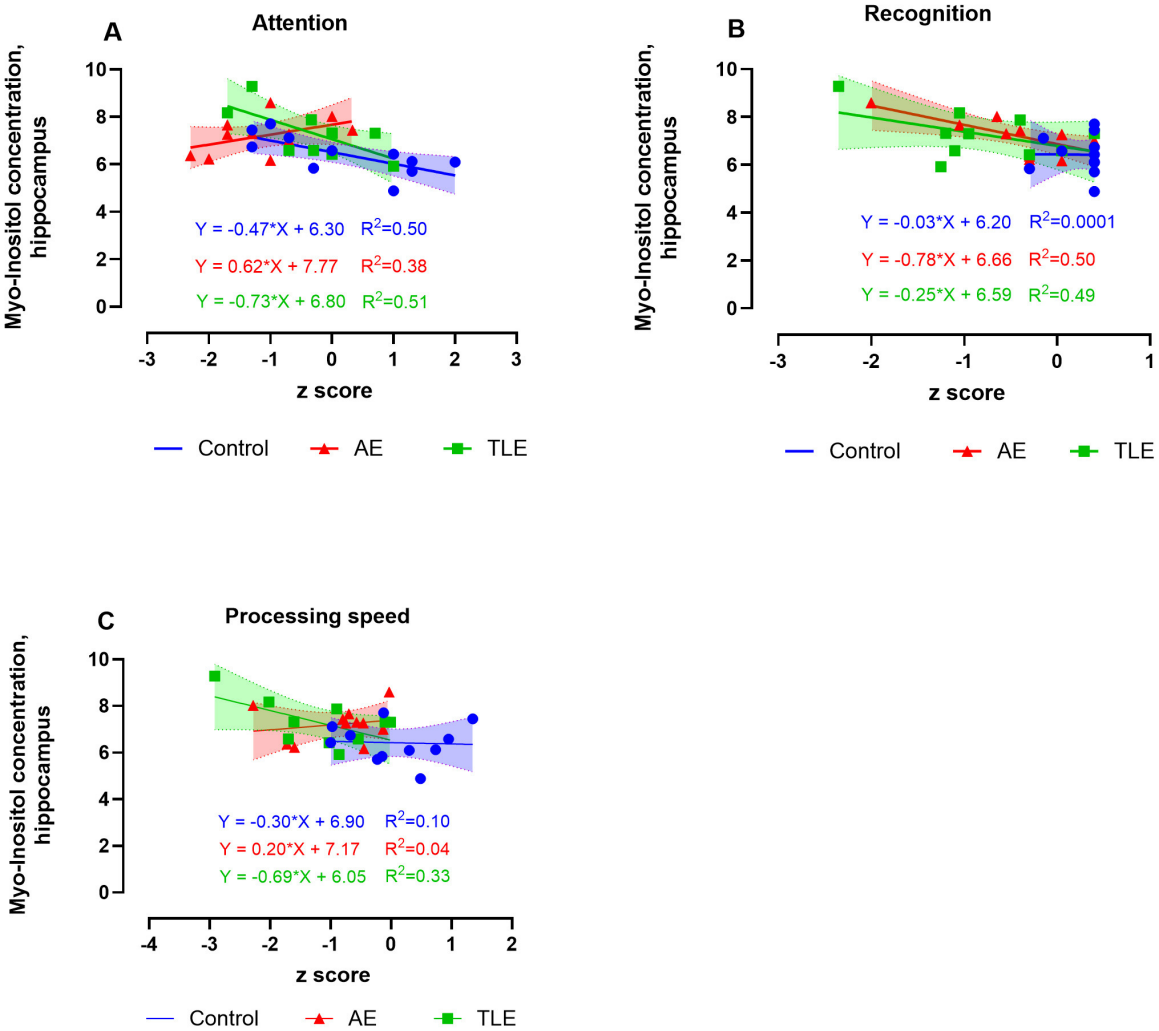
Relationship between the ^1^H-MRS measures of hippocampal myo-inositol (M-Ins) and cognitive performance in patients with autoimmune encephalitis (AE), temporal lobe epilepsy (TLE), and control subjects. The levels of M-ins positively correlated with attention **(A)** and negatively correlated with recognition **(B)**. The M-Ins concentrations negatively correlated with attention and processing speed in TLE **(C)**. The data are concentrations of metabolite (μmol/g) and mean standardized z scores for each cognitive domain.

The hippocampal concentrations of M-Ins positively correlated with attention and negatively correlated with recognition measures in AE such that lower and higher levels of M-Ins, respectively were accompanied by worse performance on these tasks (*R*^2^ = 0.38, *p* = 0.04 and *R*^2^ = 0.50, *p* = 0.02, respectively; Figures 5A, B; Supplementary Table 6). The M-Ins concentrations have negatively correlated with attention and processing speed in TLE (*R*^2^ = 0.51; *p* = 0.009 and *R*^2^ = 0.33; *p* = 0.05, respectively; Figure 5C; Supplementary Table 6). Curiously, the hippocampal M-Ins concentrations negatively correlated with attention and positively correlated with memory scores in control subjects (*R*^2^ = 0.50, *p* = 0.02 and *R*^2^ = 0.42, *p* = 0.03; Supplementary Table 6). There were no correlations between tCr, tCho, or M-Ins and other cognitive measures in AE, TLE, or control participants.

In summary, we established that higher concentrations of M-ins in the hippocampus correlate with worse measures of attention and better recognition scores in patients with post-acute AE. On the other hand, high concentrations of tCho, tCr, and M-Ins selectively correlated with worse performance on attention, processing speed, language, memory, and learning tasks in TLE.

## 4 Discussion

In the present study, we applied the *in vivo* ^1^H-MRS to map the metabolite profiles of cognitively impaired patients with post-acute AE and compared them to those in patients with normal control subjects and patients with non-lesional TLE. Consistent with previous reports (4, 6, 12), patients with AE demonstrated a wide range of cognitive impairments, including attention, processing speed, language, visuospatial skills, learning, and recognition; the first three domains were particularly affected. Similarly, patients with non-lesional TLE have demonstrated impairment in multiple cognitive domains. Using the ^1^H-MRS, we showed that when compared to the control subjects, the concentrations of M-Ins were higher in both patient groups while concentrations of tCho and tCr were higher in the TLE group; these changes were specific to the hippocampus. The higher concentrations of M-ins correlated with worse recognition and better attention scores in AE. On the other hand, higher concentration M-Ins, tCr, and tCho have selectively correlated with worse performance on the tasks of attention, processing speed, language, and memory in TLE. These findings suggest that patients with AE and TLE have unique neuropsychological profiles and distinct metabolic spectra in the hippocampus. Further, our findings indicate that glial cell activation in the hippocampus contributes to the pathogenesis of cognitive failure in chronic AE and TLE.

One potential emerging mechanism of cognitive loss in chronic central nervous system (CNS) disorders is metabolic dysfunction which can be monitored dynamically using the ^1^H-MRS (35). We have established a new methodology of ^1^H-MRS by using a semiLASER sequence at the high field of 3 T which allowed us to enhance spectral quality, increase signal-to-noise ratio, and improve localization accuracy of brain metabolites compared to the conventional sequences. These attributes permitted precise quantification of metabolites in the hippocampus, a small and deep brain structure with a less uniform magnetic field (16). Our study achieved excellent spectral quality, characterized by increased SNRs and narrow linewidths, resulting in low CRLBs that permitted reliable quantification of metabolites with overlapping resonances or low concentrations, such as Glu and GSH. Additionally, the within-VOI tissue composition and average brain tissue water content were well aligned with previous reports, demonstrating consistent within-VOI tissue composition and accurate MRS voxel placement among groups (28, 36). Moreover, CSF correction, approximately 3% in the hippocampus and 1% in the cortex, had only a minor impact on metabolite quantification. Finally, the mean metabolite concentrations of tNAA, tCho, tCr, and M-ins in the control subjects in our study were consistent with those reported in previous studies (28, 36, 37).

To the best of our knowledge, this study was the first to systematically evaluate the region-specific spectra of metabolites in a cohort of AE patients. The M-Ins concentration tended to increase in the hippocampus during the post-acute AE phase which was consistent with a report of a serial ^1^H-MRS imaging in a patient with anti-NMDAR encephalitis (38). This metabolite serves several critical functions in the brain, including the regulation of cellular osmolarity, particularly during exposure to the hypotonic or hypertonic environments (39, 40). Furthermore, M-Ins acts as a precursor for the synthesis of the membrane lipid phosphatidylinositol 4,5-bisphosphate (PIP2) and more complex inositol phosphates that are supporting signal transduction and cellular signaling (41). A portion of M-Ins is synthesized *de novo* in astrocytes from glucose. Additionally, astrocytes express the sodium-dependent myo-inositol transporters SMIT1 and SMIT2, which regulate the myo-inositol uptake (42). Brain M-Ins levels are increased in the setting of acute hyperglycemia and chronic metabolic syndrome (43). Elevated brain levels of M-Ins have also been observed in AD. M-Ins is considered a glial marker due to its association with gliosis and inflammatory states (25, 44). Thus, our study provides indirect evidence of glial cell involvement in the pathophysiology of AE with cognitive loss. Given that our patients with AE had infrequent seizures, the observed increase in M-Ins was not likely caused by seizure activity. Future longitudinal studies with serial measurement using ^1^H-MRS can shed light on the utility of M-ins for monitoring the clinical recovery in transition to a latent phase of AE and its potential application as a disease biomarker.

Previous studies suggested that signal change from M-ins in the hippocampus of patients with epilepsy may reflect the direct effects of seizures and those of anti-seizure medications (ASMs) (45, 46). These findings have prompted us to compare the metabolic spectra in AE to those in non-lesional TLE with ongoing seizures. We found that despite the qualitative increase in M-Ins signal in TLE compared to the control subjects, the differences in metabolite concentrations only showed a trend. This could be due to larger than expected variability in the concentrations of M-Ins in this group requiring a larger sample size. The M-Ins signal in TLE also did not differ from that in AE. Since the TLE patients had higher seizure burden and ASM usage compared to AE patients, the augmented M-Ins signal in AE was not likely caused by seizure activity or ASM effects. Other studies in AE patients using the whole brain PET have shown hypermetabolism involving the mesial temporal lobe and widespread hypometabolism in the cortical areas (47). We did not detect any changes in the metabolite concentrations in the cortex. This could be explained by the differences in techniques and the timing of assessment with respect to the onset of AE. Indeed, FDG PET has normalized during clinical recovery in patients with anti-NMDAR encephalitis (48).

Quantitative changes in the hippocampal tNAA or tNAA/Cr ratio, the markers of neuronal loss, have been linked to cognitive deterioration in MCI, AD, and TLE (49, 50). Changes in other metabolites, such as Cr, Cho, and M-Ins have been related to cognitive loss in MCI, AD, and epilepsy indicating that metabolic compromise and immune activation disrupt cognition (49, 51). Our findings suggest that both AE and TLE are associated with elevated brain M-Ins; however, the data indicate that AE is primarily characterized by quantitative increase in M-Ins, whereas TLE shows more pronounced alterations in tCr and tCho. While M-Ins is widely recognized as an astrocytic marker, tCho has been linked to several processes, including membrane turnover, myelin integrity, microglial polarization, and astrocytic anti-inflammatory responses (52, 53). Elevated brain tCho levels have also been reported in traumatic brain injury and various neurodegenerative disorders (52, 53). These findings raise the intriguing possibility that, although astrogliosis is a common feature in both AE and TLE, it may occur through distinct pathophysiological mechanisms unique to each condition.

The positive correlation of M-Ins level with attention scores and tCr level with processing speed in AE in our study agrees with these findings and suggests a modulatory effect of hippocampal metabolites on these cognitive functions. Consistent with previous reports (54), we found a negative correlation of the hippocampal M-Ins, Cho, and Cr concentrations with the scores reflecting attention, processing speed, language, and memory functions in TLE. Along with the hippocampus, these cognitive domains are supported by other regions of the temporal, parietal, and frontal cortex, suggesting a much broader influence of metabolic disruption in the hippocampus on these cognitive functions in TLE. Taken together with previous studies, our current findings suggest that dysregulation of brain M-Ins may be a common feature of both AE and TLE-associated cognitive loss.

Some limitations of our study are worth noting. The main limitation was an inability to control the timing of brain imaging with respect to AE symptoms and initiation of immunotherapy. As such, current findings represent a cross-sectional assessment of metabolism in AE patients tested at different stages of post-acute recovery. In *post hoc* analyses of M-Ins and in comparisons of Glu levels across the three groups, we observed a trend toward increased metabolite levels. It is likely that the small sample size contributed to the inability to detect statistically significant differences. Due to this limitation, we were also unable to examine brain metabolite changes in specific AE subtypes or assess the impact of seizure lateralization in TLE. These factors may limit the generalizability of our findings. Future studies, preferably conducted through multicenter collaborations, will be necessary to address these important questions. Despite the different pathophysiology of acute AE, chronic cognitive impairment in AE appears to have a shared footprint. In patients with TLE, the effects of language dominance on cognitive function could not be assessed due to the small cohort size. Finally, our study was conducted at the level 4 epilepsy center which attracts the patients with the most severe AE and TLE. Therefore, the generalization of these findings to the broader patient populations in other clinical settings may be limited.

## 5 Conclusions

We found that despite similar subjective complaints of cognitive impairment, patients with AE and TLE have demonstrated distinct profiles of metabolites associated with neuronal dysfunction and glial activation in the hippocampus. The disease-specific metabolite changes have selectively modulated the cognitive function in AE and TLE.

## Data Availability

The raw data supporting the conclusions of this article will be made available by the authors, without undue reservation.
