# Supplementary material for: Hippocampal dysmetabolism contributes to cognitive loss in autoimmune encephalitis and focal temporal epilepsy
Source: Front Neurol. 2025 Aug 14;16:1597928. doi: 10.3389/fneur.2025.1597928 (PMC12391106; doi:10.3389/fneur.2025.1597928)
Supplement: Supplementary file 1 [file Supplementary_file_1.docx]

**Supplemental Table 1.** Checklist of items for the Strengthening the Reporting of Observational Studies in Epidemiology (STROBE) statement.

|  | Item No. | Recommendation | Page  No. |
| --- | --- | --- | --- |
| **Title and abstract** | 1 | (a) Indicate the study’s design with a commonly used term in the title or the abstract | 1 |
|  |  | (b) Provide in the abstract an informative and balanced summary of what was done and what was found | 2-3 |
| Introduction | | | |
| Background/rationale | 2 | Explain the scientific background and rationale for the investigation being reported | 5-6 |
| Objectives | 3 | State specific objectives, including any pre-specified hypotheses | 6 |
| Methods | | | |
| Study design | 4 | Present key elements of study design early in the paper | 6-8 |
| Setting | 5 | Describe the setting, locations, and relevant dates, including periods of recruitment, exposure, follow-up, and data collection | 6-8 |
| Participants | 6 | (*a*) *Cohort study*—Give the eligibility criteria, and the sources and methods of selection of participants. Describe methods of follow-up | 6-7 |
|  |  | (*b*) *Cohort study*—For matched studies, give matching criteria and number of exposed and unexposed | NA |
| Variables | 7 | Clearly define all outcomes, exposures, predictors, potential confounders, and effect modifiers. Give diagnostic criteria, if applicable | 6-8, 11 |
| Data sources/measurement | 8 | For each variable of interest, give sources of data and details of methods of assessment (measurement). Describe comparability of assessment methods if there is more than one group | 11 |
| Bias | 9 | Describe any efforts to address potential sources of bias | 8 |
| Study size | 10 | Explain how the study size was arrived at | 7 |

Continued on next page

| Quantitative variables | 11 | Explain how quantitative variables were handled in the analyses. If applicable, describe which groupings were chosen and why | 6.9 |
| --- | --- | --- | --- |
| Statistical methods | 12 | (*a*) Describe all statistical methods, including those used to control for confounding | 9 |
|  |  | (*b*) Describe any methods used to examine subgroups and interactions | 9 |
|  |  | (*c*) Explain how missing data were addressed | 9,11 |
|  |  | (*d*) *Cohort study*—If applicable, explain how loss to follow-up was addressed | N/A |
|  |  | (*e*) Describe any sensitivity analyses | NA |
| Participants | 13 | (a) Report numbers of individuals at each stage of study—e.g., numbers potentially eligible, examined for eligibility, confirmed eligible, included in the study, completing follow-up, and analyzed | 11 |
|  |  | (b) Give reasons for non-participation at each stage | N/A |
|  |  | (c) Consider use of a flow diagram | NA |
| Descriptive data | 14 | (a) Give characteristics of study participants (e.g., demographic, clinical, social) and information on exposures and potential confounders | 11-12; Table 1 |
|  |  | (b) Indicate number of participants with missing data for each variable of interest | 11 |
|  |  | (c) *Cohort study*—Summarize follow-up time (e.g., average and total amount) | 12, Table 1 |
| Outcome data | 15 | *Cohort study*—Report numbers of outcome events or summary measures over time | 10, Table 1, Suppl. Tables 5,6 |
| Main results | 16 | (*a*) Give unadjusted estimates and, if applicable, confounder-adjusted estimates and their precision (e.g., 95% confidence interval). | 14-15; Suppl. Tables 5,6 |
|  |  | (*b*) Report category boundaries when continuous variables were categorized | NA |
|  |  | (*c*) If relevant, consider translating estimates of relative risk into absolute risk for a meaningful time period | NA |

**Supplemental Table 2.** Neuropsychological tests with the corresponding cognitive domains

| Test, Subtests, and Tasks | Assigned Domain |
| --- | --- |
|  |  |
| Wechsler Adult Intelligence Scale (WAIS-IV)^1^  *Digit Span Forward*  *Digit Span Backward*  *Reliable Digit Span*  *Letter Number Sequencing* | Attention Executive Functioning Validity/Effort Executive Functioning |
| Dot Counting Test (DCT)^2^ | Validity/Effort |
| Golden Stroop (Stroop) ^3,4^  *Word Reading*  *Color Naming*  *Color Word Reading* | Processing Speed Processing Speed Executive Functioning |
| Trail Making Test (TMT)^5^   *Form A*  *Form B* | Processing Speed Executive Functioning |
| Symbol Digit Modalities Test (SDMT)^6^ | Processing Speed |
| Boston Naming Test (BNT)^4^ | Language |
| Controlled Oral Word Association (COWAT)^4^ | Language |
| Judgement of Line Orientation (JLO)^4,7^ | Visuospatial |
| Rey-Osterrith Complex Figure copy (RCFT)^8^ | Visuospatial |
| Brief Visuospatial Memory Test – Revised (BVMT-R)^9^  *Trials 1-3 Total*  *Delayed Recall Trial*  *Recognition Trial* | Learning Memory Recognition |
| Hopkins Verbal Learning Test Revised (HVLT-R)^10^  *Trials 1-5 Total*  *Delayed Recall Trial*  *Recognition Trial* | Learning Memory Recognition |
| Self-Report Measures  *Generalized Anxiety Disorder-7 (GAD-7)*^11^  *Patient Health Questionnaire-9 (PHQ-9)*^12^  *Pittsburgh Sleep Quality Index (PSQI)*^13^ | Mood (Anxiety) Mood (Depression Sleep |

**References**

1. Wechsler D. *WAIS-IV: Wechsler Adult Intelligence Scale*. Psychological Corporation; 2008.

2. Boone KB, Lu P, Herzberg D. The Dot Counting Test^TM^(DCT^TM^). *Torrance (CA): Western Psychological Services (WPS)*. Published online 2002.

3. Golden C, Freshwater S. *The Stroop Color and Word Test: A Manual for Clinical and Experimental Uses*. Stoelting; 2002.

4. Ivnik RJ, Malec JF, Smith GE, Tangalos EG, Petersen RC. Neuropsychological tests’ norms above age 55: COWAT, BNT, MAE token, WRAT-R reading, AMNART, STROOP, TMT, and JLO. *The Clinical Neuropsychologist*. 1996;10(3):262-278. doi:10.1080/13854049608406689

5. Heaton RK. *Revised Comprehensive Norms for an Expanded Halstead-Reitan Battery: Demographically Adjusted Neuropsychological Norms for African American and Caucasian Adults, Professional Manual*. Psychological Assessment Resources; 2004.

6. Smith A. Symbol digit modalities test. *The clinical neuropsychologist*. Published online 1973. Accessed September 21, 2024. https://psycnet.apa.org/doiLanding?doi=10.1037/t27513-000

7. Benton A, Hamsher K, Varney N, Spreen Ö. Contri-butions to neuropsychological assessment: A clinical manual New York: Oxford blniversity Press. Published online 1983.

8. Meyers JE, Meyers KR. Rey complex figure test under four different administration procedures. *The Clinical Neuropsychologist*. 1995;9(1):63-67. doi:10.1080/13854049508402059

9. Benedict RHB. Brief Visuospatial Memory Test-Revised. *Psychological Assessment Resources, Inc*. Published online 1997.

10. Brandt J. Hopkins verbal learning test. *Clinical Neuropsychologist*. Published online 2001. Accessed September 21, 2024. https://psycnet.apa.org/getdoi.cfm?doi=10.1037/t49859-000

11. Spitzer RL, Kroenke K, Williams JB, Löwe B. A brief measure for assessing generalized anxiety disorder: the GAD-7. *Archives of internal medicine*. 2006;166(10):1092-1097.

12. Kroenke K, Spitzer RL, Williams JBW. The PHQ-9: Validity of a brief depression severity measure. *J Gen Intern Med*. 2001;16(9):606-613. doi:10.1046/j.1525-1497.2001.016009606.x

13. Buysse DJ, Reynolds III CF, Monk TH, Berman SR, Kupfer DJ. The Pittsburgh Sleep Quality Index: a new instrument for psychiatric practice and research. *Psychiatry research*. 1989;28(2):193-213.

**Supplemental Table 3.** Demographic and clinical characteristics of patients with autoimmune encephalitis (AE).

| **Patient ID** | **A003** | **A004** | **A005** | **A006** | **A007** | **A008** |
| --- | --- | --- | --- | --- | --- | --- |
| **Age, sex** | 27, F | 27, F | 26, F | 28, F | 66, M | 37, F |
| **Handedness** | R | R | R | R | R | R |
| **Duration, months** | 50 | 48 | 62 | 60 | 154 | 54 |
| **Syndrome type** | Anti-NMDA | Anti-NMDA | Anti-NMDA | Anti-NMDA | Anti-LGI-1 | Anti-GAD 65 |
| **Presenting symptoms** | Behavior and personality changes, confusion,  dysarthria, asymmetric GTCs | Auditory hallucinations, orolingual dyskinesia, myoclonus, catatonia | New onset GTC, confusion, catatonia, hyperactivity, impulsivity | Left hand twitching, chin quivering, left-sided numbness | Confusion, facial contortions (suspected FBDS) | Memory loss, disorientation, deja vu |
| **CSF findings on presentation** | WBC 19 (L 75%), IgG index 1.58 and synthesis rate 19.1, anti-NMDA IgG (1:28) | WBC 25, protein 55, IgG synthesis rate 17, OCB >10, anti-NMDA IgG | WBC 72 (L 79%),  OCB 7, anti-NMDA  IgG (1:64) | WBC 21 (L 49%) Anti-NMDA IgG | Protein 92 | WBC 9 (L 95%), anti-GAD 65 IgG (17.7 nmol/L) |
| **Serum studies** | Anti-NMDA IgG (1:10) | Anti-NMDA IgG (1:160) | Anti-NMDA IgG + | Anti-MOG IgG | Anti-LGI1 IgG |  |
| **EEG findings on presentation** | R hemispheric LRDA | GRDA, diffuse slowing | No lateralizing or epileptiform features | N/A | Bitemporal epileptiform discharges | Clinical and subclinical L temporal seizures |
| **Seizure count/ year** | 0-2^a^ | 0 | 0 | 0 | 0^b^ | 0^b^ |
| **ASMs, count** | 1 | 0 | 0 | 0 | 2 | 1 |
| **ASMs, names** | BRV | None | None | None | LEV, LCM | LEV |
| **Immunotherapy** | Rituximab | Rituximab, IVIG | None | IVIG | IVIG | Rituximab, IVIG |
| **Non-ASM, count** | 0 | 5 | 0 | 1 | 2 | 2 |

Continued on next page

| **Patient ID** | **A009** | **A017** | **A019** | **A020** | **A040** | **A042** |
| --- | --- | --- | --- | --- | --- | --- |
| **Age, sex** | 37, M | 60, M | 25, F | 52, F | 58, M | 21, F |
| **Handedness** | R | R | L | R | R | L |
| **Duration, months** | 43 | 204 | 3 | 35 | 24 | 1 |
| **Syndrome type** | Unknown | Anti-GAD 65 | Unknown/ NORSE | Unknown/NORSE | Unknown/NORSE | Anti-NMDA |
| **Presenting symptoms** | Convulsions,  memory difficulty | Headache, bilateral peripheral vision loss, bilateral arm paresthesia | Personality changes, headache, abdominal pain, GTC | Fluctuating confusion, mutism | Aphasia, unresponsiveness, staring, GTC | Cognitive and neuropsychiatric changes |
| **CSF findings on presentation** | WBC 26 (N 93%),  Protein 48, OCB 1 | Protein 73, anti-GAD 65 IgG (108 mmol/L) | Increased IL-6,  IL-12, IL-17 | Protein 92,  glucose 196,  increased IL-6 | Protein 205, IgG 10.1, IgG synthesis rate 18.6 | WBC 12 (L 95%),  OCB >10, anti-NMDA IgG (1;128) |
| **Serum studies** | Normal | Anti-GAD 65 IgG (51) | Normal | Normal | Normal | Normal |
| **EEG findings on presentation** | Diffuse slowing | Normal | Multiple electrographic and FBTC seizures with L>R hemispheric onset | Generalized nonconvulsive status epilepticus | R centrotemporal LRDA, seizures | Slowing in L hemisphere, LRDA |
| **Seizure count/ year** | 5 | 0 | 0 | 5 | 0 | 0 |
| **ASMs, count** | 3 | 3 | 5 | 4 | 1 | 1 |
| **ASMs, names** | LEV, LCM, LTG | PGB, VPA, CZP | CLB, LEV, LCM, PHT, PER | CLB, LEV, LCM, PGB | PGB | LEV |
| **Immunotherapy** | IVIG | IVIG, methotrexate | Anakinra | IVIG, Anakinra | None | Rituximab |
| **Non-ASMs, count** | 1 | 8 | 1 | 11 | 3 | 0 |

Abbreviations: R, right; L, left; anti-NMDA, anti-N-methyl D-aspartate; anti-LGI-1, anti-leucine glioma inactivated protein 1; anti-GAD-65, anti-glutamic acid decarboxylase; anti-MOG, anti-myelin oligodendrocyte glycoprotein, NORSE, new onset refractory status epilepticus; ASM, antiseizure medication; GTC, generalized tonic-clonic seizure, WBC, white blood cell; OCB, oligoclonal bands; IL, interleukin, L, lymphocytes; M, monocytes; LRDA, lateralized rhythmic delta activity; GRDA, generalized rhythmic delta activity; FBDS, facial brachial dystonic; FBTC, focal to bilateral tonic clonic, BRV, brivaracetam; LEV, levetiracetam; LCM, lacosamide; IVIG, intravenous immunoglobulin. ^a, b^ The findings on research MRI imaging, temporal lobe. ^a^ Decreased size of the left amygdala measuring at the 13% normal percentile; ^b^ Bilateral hippocampal volume loss and increased T2 signal.

**Supplemental Table 4.** Demographic and clinical characteristics of patients with temporal lobe epilepsy (TLE).

| **Patient ID** | **Age,**  **sex** | **Duration, months** | **Handedness** | **Refractory status** | **Seizure types** | **Seizure laterality** | **Seizure count/**  **year** | **ASMs,**  **count** | **ASM names** | **Non-ASMs,**  **count** |
| --- | --- | --- | --- | --- | --- | --- | --- | --- | --- | --- |
| A027 | 64, F | 12 | R | N | FBTCS, FIAS | L | <1 | 2 | LTG, LEV | 18 |
| A029^a^ | 62, M | 60 | R | Y | FBTCS, FIAS | L | 4 | 3 | BRV, LCM, LTG | 7 |
| A030^b^ | 65, F | 408 | R | Y | FBTCS, FIAS | L | 6 | 2 | BRV, LTG | 5 |
| A031^c^ | 41, M | 120 | R | Y | FBTCS, FIAS | L | 12 | 4 | LEV, OXC, CNB, CBD | 3 |
| A032 | 31, F | 72 | R | N | FIAS | BL | 0 | 2 | CLB, LEV | 8 |
| A034 | 31, F | 60 | R | Y | FBTCS, FIAS, FAS | L | 48 | 3 | CLB, LCM, LEV | 6 |
| A035 | 46, F | 36 | R | Y | FBTCS, FIAS | L | 48 | 3 | CNB, LCM, LEV | 14 |
| A036 | 48, F | 108 | L | Y | FBTCS, FIAS | R | 48 | 3 | LCM, TPM, PER | 5 |
| A037 | 48, M | 168 | R | Y | FBTCS, FAS | R | 240 | 3 | CLB, LTG, LEV | 5 |
| A038 | 22, F | 156 | R | Y | FIAS | L | 12 | 4 | CLB, CZP, LEV, OXC | 1 |
| A041 | 20, M | 52 | L | Y | FBTCS, FIAS, FAS | L | 144 | 4 | CLB, CZP, LEV, OXC | 0 |
| A043^d^ | 62, F | 180 | R | N | FBTCS, FIAS | R | 0 | 2 | LCM, PGB | 8 |

Abbreviations: M, male; F, female; R, right; L, left; BL, bilateral; ASM, antiseizure medication; FBTCS, focal to bilateral tonic clonic seizure; FIAS, focal impaired awareness seizure; FAS, focal aware seizure; LTG, lamotrigine; LEV, ;levetiracetam; BRV, brivaracetam; LCM, lacosamide; OXC, oxcarbazepine, CNB, cenobamate; CBD, cannabidiol; CLB, clobazam; TPM, topiramate; PER, perampanel; CZP, clonazepam, PGB, pregabalin. ^a, b,c,d^ The findings on research MRI imaging, temporal lobe. ^a^ Focal area of contrast enhancement in the posterior lateral left temporal lobe; ^b^ Suspected left hippocampal cyst, right hippocampal cyst versus perivascular space; ^c^ Mild susceptibility hypo-intensities in the left temporal lobe; ^d^ Bilateral anterior temporal meningoceles.

**Supplemental Table 5.** Concentrations of metabolites (µmol/g, mean ± SEM) in the hippocampus and cortex of patients with autoimmune encephalitis (AE), temporal lobe epilepsy (TLE) and control subjects. The data are. tNAA, N-acetyl aspartate; tCho, choline-containing compounds; tCr, creatine and phosphocreatine; Glu, glutamate; M-Ins, *myo*-inositol; GSH, glutathione. ∗∗ *p* < 0.01 vs. control, post-hoc test; ∗ *p* < 0.05 vs. control, post-hoc test; # 0.05 ≤ *p* < 0. 1 vs. trend for a change without significance vs. control, post-hoc tests.

Hippocampus

| Metabolite | Control | AE | TLE |
| --- | --- | --- | --- |
| tNAA | 7.43 ± 0.42 | 7.16 ± 0.52 | 7.60 ± 0.46 |
| tCho | 1.94 ± 0.16 | 2.01 ± 0.18 | 2.14 ± 0.23 * |
| tCr | 6.05 ± 0.43 | 6.30 ± 0.39 | 6.56 ± 0.36 ** |
| Glu | 5.92 ± 0.63 | 5.97 ± 0.68 | 6.15 ± 0.79 |
| M-Ins | 6.49 ± 0.82 | 7.19 ± 0.72 # | 7.04 ± 1.05 # |
| GSH | 1.15 ± 0.35 | 1.13 ± 0.29 | 1.32 ± 0.33 |

Cortex

| Metabolite | Control | AE | TLE |
| --- | --- | --- | --- |
| tNAA | 8.97 ± 0.37 | 8.99 ± 0.69 | 8.55 ± 0.76 |
| tCho | 1.80 ± 0.30 | 2.04 ± 0.46 | 2.11 ± 0.22 |
| tCr | 5.98 ± 0.29 | 6.19 ± 0.44 | 6.02 ± 0.56 |
| Glu | 6.39 ± 0.80 | 5.96 ± 1.20 | 5.98 ± 0.61 |
| M-Ins | 5.48 ± 0.63 | 5.92 ± 1.50 | 5.62 ± 1.20 |
| GSH | 1.22 ± 0.21 | 1.27 ± 0.21 | 1.28 ± 0.14 |

**Supplemental Table 6.** Correlations between the relevant metabolite concentrations in the hippocampus and cognitive measures in patients with autoimmune encephalitis (AE) and temporal lobe epilepsy (TLE). The relationships between the mena concentrations of the metabolites in the left and right hippocampus and z-scores in each cognitive domain were assessed using linear regression; the strength of associations was determined using the Pearson r; CI, 95% confidence interval. ∗ *p* < 0.05; ∗∗ *p* < 0.01; simple linear regression analysis.

Total choline (tCho)

| Cognitive Measures | Control, r (CI) | AE, r (CI) | TLE, r (CI) |
| --- | --- | --- | --- |
| Attention | -0.30 (-0.76; 0.36) | 0.28 (-0.39; 0.73) | **-0.80 (-0.94; -0.42) ^**^** |
| Processing speed | -0.05 (-0.63; 0.56) | -0.02 (-0.61; 0.59) | **-0.75 (-0.93; -0.31) ^**^** |
| Executive function | -0.14 (-0.68; 0.50) | -0.12 (-0.67; 0.52) | -0.51 (-0.84; 0.09) |
| Language | 0.41 (-0.26; 0.81) | -0.10 (-0.69; 0.56) | **-0.66 (-0.90; -0.14) ^*^** |
| Memory | -0.21 (-0.72; 0.44) | -0.35 (-0.78; 0.32) | 0.56 (-0.86; 0.03) |
| Visuospatial | 0.04 (-0.61; 0.65) | 0.15 (-0.50; 0.69) | -0.54 (-0.85; 0.05) |
| Learning | -0.51 (-0.85; 0.13) | 0.25 (-0.74; 0.41) | -0.53 (-0.84; 0.07) |
| Recognition | 0.11 (-0.52; 0.67) | -0.21 (-0.72; 0.45) | -0.37 (-0.78; 0.30) |

Total creatine (tCr)

| Cognitive Measures | Control, r (CI) | AE, r (CI) | TLE, r (CI) |
| --- | --- | --- | --- |
| Attention | -0.51 (-0.85; 0.13) | -0.41 (-0.82; 0.25) | **-0.81 (-0.94; -0.44) ^**^** |
| Processing peed | -0.07; (-0.65; 0.55) | **0.62 (0.02; 0.89) ^*^** | -0.50 (-0.83; 0.10) |
| Executive function | -0.33 (-0.77; 0.33) | -0.01 (-0.61; 0.59) | -0.38 (-0.79; 0.24) |
| Language | 0.01 (-0.59; 0.61) | 0.50 (-0.19; 0.86) | -0.48 (-0.83; 0.13) |
| Memory | 0.27 (-0.39; 0.75) | -0.26 (-0.74; 0.40) | **-0. 73 (-0.91: -0.27) ^**^** |
| Visuospatial | 0.03 (-0.58; 0.62) | 0.36 (-0.30; 0.79) | -0.28 (-0.73; 0.35) |
| Learning | 0.08 (-0.55; 0.65) | -0.21 (-0.72; 0.44) | **-0.59 (-0.87: -0.02) ^*^** |
| Recognition | 0.39 (-0.27; 0.80) | -0.001 (-0.60; 0.60) | -0.53 (-0.85; 0.06) |

*Myo*-inositol (M-Ins)

| Cognitive Measures | Control, r (CI) | AE, r (CI) | TLE, r (CI) |
| --- | --- | --- | --- |
| Attention | **-0.70 (-0.92; -0.18) ^*^** | **0.62 (0.03; 0.89) ^*^** | **-0.71 (-0.91; -0.24) ^**^** |
| Processing speed | -0.31 (-0.75; 0.32) | 0.19 (-0.46; 0.71) | **-0.58 (-0.86; -0.004) ^*^** |
| Executive function | 0.21 (-0.45; 0.72) | -0.08 (-0.63; 0.54) | -0.21 (-0.70; 0.42) |
| Language | 0.36 (-0.30; 0.79) | 0.15 (-0.53; 0.71) | -0.47 (-0.83; 0.14) |
| Memory | **0.65 (0.08; 0.90) ^*^** | -0.24 (-0.73; 0.43) | 0.47 (-0.15; 0.82) |
| Visuospatial | -0.48 (-0.84; 0.17) | 0.16 (-0.49; 0.69) | -0. 38 (-0.78; 0.25) |
| Learning | -0.01 (-0.61; 0.59) | -0.12 (-0.67; 0.51) | -0.41 (-0.79; 0.22) |
| Recognition | -0.30 (-0.76; 0.36) | **-0.71 (-0.92; -0.18) ^*^** | -0.22 (-0.71; 0.40) |
