# Supplementary material for: Hippocampal dysmetabolism contributes to cognitive loss in autoimmune encephalitis and focal temporal epilepsy
Source: Front Neurol. 2025 Aug 14;16:1597928. doi: 10.3389/fneur.2025.1597928 (PMC12391106; doi:10.3389/fneur.2025.1597928)
Supplement: Supplementary file 2 [file Data_Sheet_1.docx]

**Supplemental Figure 1.** Relationship between the ^1^H-MRS measures of hippocampal total choline (tCho) and cognitive performance in patients with autoimmune encephalitis (AE), temporal lobe epilepsy (TLE), and control subjects. The levels of tCho inversely corrected with the standardized z scores in the measures of attention **(A)**, processing speed **(B)**, and language **(C)** while no significant correlations were observed in AE and control subjects. The data are concentrations of metabolite (µmol/g) and mean standardized z scores for each cognitive domain.

**Supplemental Figure 2.** Mean Cramer-Rao lower bounds (CRLBs) of the metabolites in the hippocampus **(A)** and cortex **(B)**. The data are mean ± standard deviation of the mean (SD).

Glutamine (Gln), glutamate (Glu), glutathione (GSH), myo-Inositol (M-Ins), total choline (tCho), total creatine (tCr), and total N-acetyl aspartate and N-acetyl–aspartyl–glutamate (tNAA).
