# Supplementary figures and images for: Hippocampal dysmetabolism contributes to cognitive loss in autoimmune encephalitis and focal temporal epilepsy
Source: Front Neurol. 2025 Aug 14;16:1597928. doi: 10.3389/fneur.2025.1597928 (PMC12391106; doi:10.3389/fneur.2025.1597928)

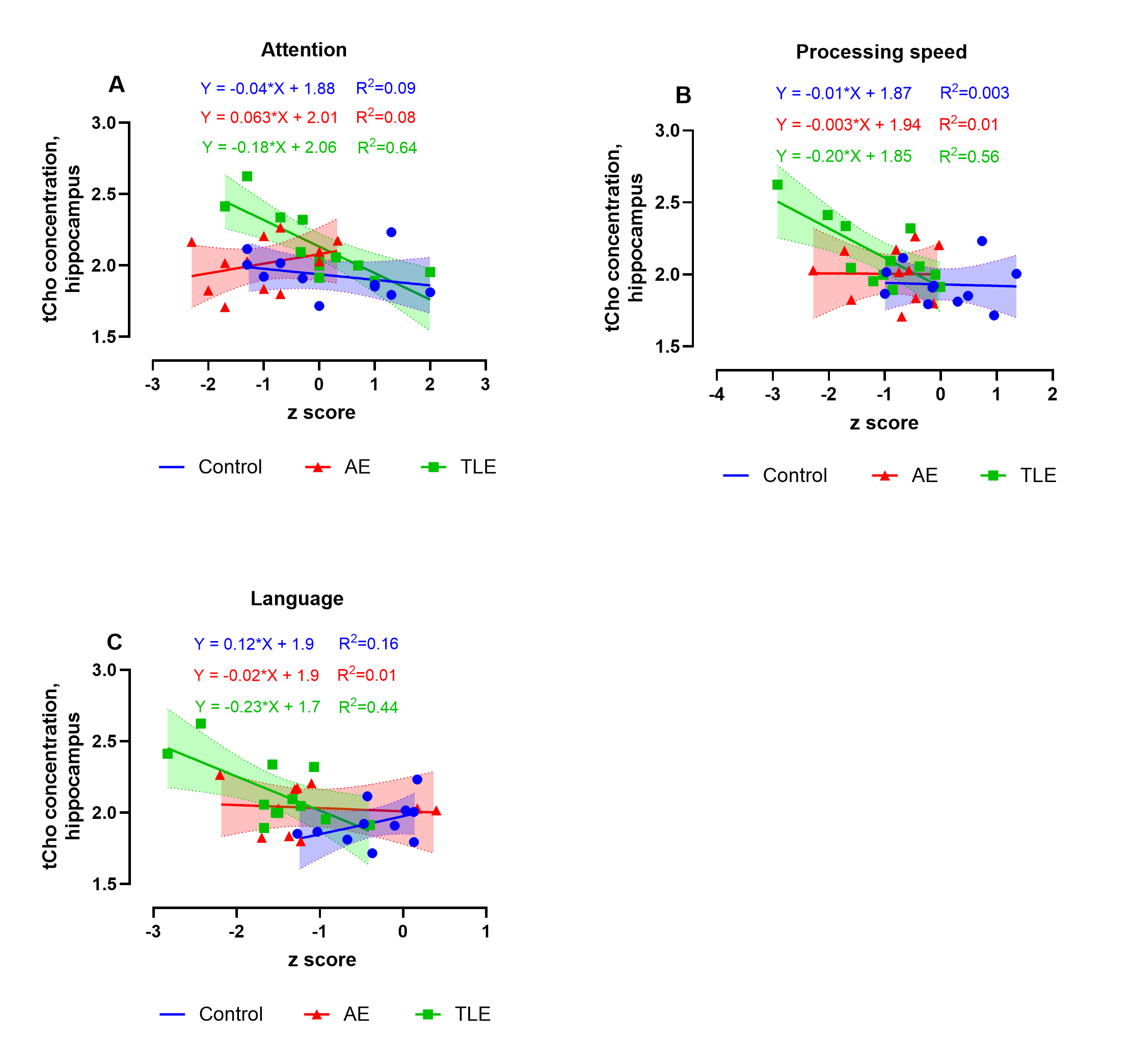

Supplement: Supplementary file 3 [file Image_1.tif]

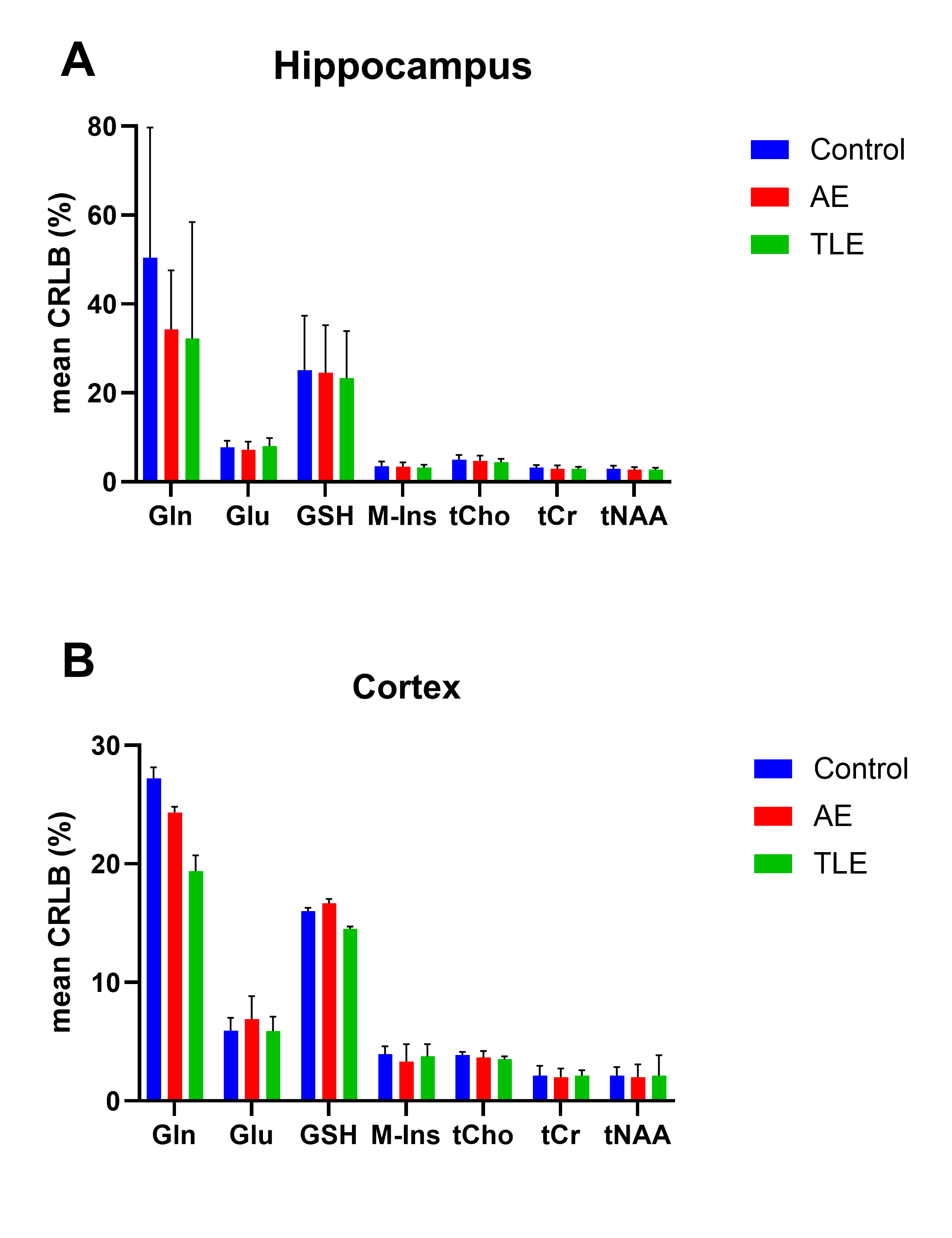

Supplement: Supplementary file 4 [file Image_2.tif]
